# The prevalence of periarticular lesions detected on magnetic resonance imaging in middle-aged and elderly persons: a cross-sectional study

**DOI:** 10.1186/s12891-016-1035-6

**Published:** 2016-04-26

**Authors:** In Je Kim, Dong Hyun Kim, Yeoung Wook Song, Ali Guermazi, Michel D. Crema, David J. Hunter, Young-Il Seo, Hyun Ah Kim

**Affiliations:** Department of Internal Medicine, Ewha Womans University Mokdong Hospital, Seoul, Republic of Korea; Department of Social and Preventive Medicine, Hallym Research Institute of Clinical Epidemiology, Hallym University, Chuncheon, Republic of Korea; Department of Internal Medicine, Seoul National University Hospital, Seoul, Republic of Korea; Department of Radiology, Boston University School of Medicine, Boston, MA USA; Department of Rheumatology, Royal North Shore Hospital and Institute of Bone and Joint Research, Kolling Institute, University of Sydney, Sydney, Australia; Department of Internal Medicine, Hallym University Sacred Heart Hospital, Anyang, Republic of Korea; Institute for Skeletal Aging, Chuncheon, Republic of Korea; Division of Rheumatology, Department of Internal Medicine, Hallym University Sacred Heart Hospital, 896, Pyongchondong, Dongan-gu, Anyang, Kyunggi-do 431-070 Republic of Korea

**Keywords:** Baker’s cyst, Anserine bursitis, Knee pain, MRI, Osteoarthritis, Periarticular lesions

## Abstract

**Background:**

Previous studies showed that among persons with radiographic knee OA, periarticular lesions were significantly more common among participants with knee pain than those without. However, data were derived mostly from persons with knee OA, and there were few normal participants without knee OA in the data analyses. The objectives of this study were to investigate the prevalence of periarticular lesions detected by magnetic resonance imaging (MRI), and to examine their prevalence according to the presence of knee pain and radiographic knee osteoarthritis (OA) in community residents in Korea.

**Methods:**

Demographic and knee pain data were obtained by questionnaire from 358 participants of the population-based Hallym Aging Study who were recruited irrespective of the presence of knee OA or pain. Radiographic evaluations consisted of weight-bearing knee anteroposterior radiographs and 1.5-T MRI scans. Periarticular lesions included prepatellar or anserine bursitis, Baker’s cyst, and tibiofibular cyst. The prevalence of each lesion in subjects with knee OA or knee pain compared to those without was examined by a chi-square test.

**Results:**

The mean age of the subjects was 72 years and 50.6 % were female. Radiographic knee OA was present in 34.5 %. The most prevalent peri-articular lesion was Baker’s cyst (27.9 %), followed by tibiofibular cyst (9.5 %). Anserine bursitis and tibulofibular cyst were more common in subjects with knee OA (17.5 % vs 2.2 % for anserine bursitis, 15.8 % vs 6.1 % for tibiofibular cyst in subjects with and without OA, respectively), while Baker’s cyst and anserine bursitis were more common in subjects with knee pain (36.3 % vs 21.8 % for Baker’s cyst, 14.4 % vs 2.5 % for anserine bursitis in subjects with and without knee pain, respectively).

**Conclusions:**

Periarticular lesions on MRI of the knee are common in middle-aged and elderly persons. Anserine bursitis and Baker’s cysts are more common in subjects with knee pain compared to those without.

## Background

Knee pain is a common musculoskeletal problem in older adults, and is closely related to radiographic osteoarthritis (OA) [1]. However, a substantial number of patients with knee pain do not exhibit radiographic OA [2]. Because recent studies suggest that knee pain is a better predictor of disability than radiographic changes, the precise determination of the causes of knee pain would be important for guiding treatment decisions [3].

Magnetic resonance imaging (MRI) allows clinicians and researchers to evaluate a range of periarticular structures not visualized by conventional radiographs, and MRI can be used for guiding therapeutic decisions. Previous studies showed that among persons with radiographic knee OA, periarticular lesions, such as anserine bursitis and iliotibial band syndrome, were significantly more common among participants with knee pain than those without and may contribute to the pain in these individuals [4]. However, the prevalence of other periarticular lesions, such as prepatellar bursitis, are apparently similar in asymptomatic and symptomatic knee OA [4], suggesting that not all inflammatory lesions in periarticular structures lead to symptoms.

In previous reports on the relationship between periarticular lesions and knee pain, data were derived mostly from persons with knee OA, and there were few normal participants without knee OA in the data analyses [4, 5]. The research questions of this cross-sectional study were: 1) what is the prevalence of periarticular lesions in the community residents according to the presence or absence of knee OA? and 2) what is the prevalence of periarticular lesions in the community residents according to the presence or absence of knee pain?

## Methods

### Subjects

The Hallym Aging Study (HAS) is a prospective cohort study investigating the health of elderly community residents in Chuncheon, a city ~120 km east of Seoul, Korea. This ongoing study began in 2004, with follow-up examinations scheduled every 3 years. Its methods have been described in detail elsewhere [6]. Briefly, 1489 subjects among 71,061 residents aged ≥ 50 years in Chuncheon were selected randomly and contacted. Participants were invited both by the telephone and mail to the study with the announcement that “This is a study evaluating general health and physical function in the elderly.” Finally 568 subjects refused to participate or were ineligible and 918 subjects finished the first wave in year 2004. Data on OA were collected from the 2^nd^ wave, which began in year 2007. Among the first wave subjects, 90 refused to participate, 13 could not be contacted, 64 had died, and 49 had moved from the area, and 702 subjects were eligible. Of them, 129 refused to undergo the OA substudy; they were significantly older than those who participated (72.3 vs. 70.4 years, *p* < 0.01), although the sex ratio was not different. Of the 573 remaining subjects, 400 were chosen randomly for knee MRIs regardless of the presence of knee pain or of radiographic knee OA. Of them, 36 declined to undergo knee MRI, three had previously undergone bilateral total knee replacements, and three had other contraindications to MRI, leaving 358 subjects who underwent knee MRIs. The Ethics Committee of Hallym University approved the study protocol. Written informed consent was obtained from all study participants.

### Data collection

Demographic information was collected using a standard questionnaire. Knee pain was assessed by asking, “Did you have pain, aching, or stiffness in either of your knees in the last month?” Those who answered “yes” was considered to have knee pain. All participants were also evaluated using the Western Ontario and McMaster Universities (WOMAC) Osteoarthritis Index for evaluating pain and self-reported functional status [7]. Height (cm) and body weight (kg) were measured to the nearest 0.1 cm and 0.1 kg, respectively, with subjects wearing light clothing and barefoot, and body mass index (BMI) was calculated as the weight in kg divided by the square of the body height in meter.

### Radiographic assessment

Radiographic evaluations consisted of 14 × 17-in., anteroposterior radiographs taken during weight bearing with a semi-flexed knee. A Plexiglas frame (SynaFlexor, San Francisco, CA, USA) was used to standardize knee position according to the manufacturer’s recommendations. Each knee was graded using the Kellgren-Lawrence (K-L) grading scale [8]. Radiographic knee OA was defined as present if the participant had a radiographic K-L grade ≥ 2 in the tibiofemoral joint. All radiographs were read twice at an interval of at least 2 weeks by one reader, an academically based rheumatologist (HAK) who was unaware of the knee pain status or MRI findings. The intra-reader reproducibility of assessments was high, with weighted kappa values of 0.86 (95 % confidence interval, CI = 0.80-0.91) for the K-L grade and 0.89 (95 % CI = 0.83-0.94) for K-L grades 0-1 (non-OA) versus K-L grade ≥ 2 (OA). Images that were assigned different K-L grades at the two readings were adjudicated by consensus between the original reader and a second reader (DJH). The weighted kappa value for inter-reader reliability was 0.84 for non-OA versus OA (95 % CI = 0.67-1.01).

### MRI and evaluation of periarticular lesions

Each participant underwent an MRI of one knee. If both knees were symptomatic, the more symptomatic knee was chosen. For subjects without knee pain, the dominant knee was selected for imaging. Knee dominance was determined by the following question: “When you start to kick a ball, which foot would you use first?” MRI scans of the knee were obtained using a 1.5-T scanner (Philips, Andover, MA, USA) with a phase-array knee coil.

The imaging protocol included a sagittal 3D water-select excited cartilage three-fluid sequence (repetition time 20 ms, echo time 7.8 ms, slice thickness 3.0 mm, interslice gap 1.5 mm, field of view 150 × 150 mm, and matrix, 304 × 304), a sagittal T2-weighted fat-suppression sequence (repetition time 2789.6 ms, echo time 60 ms, slice thickness 4.0 mm, interslice gap 0.4 mm, echo spacing 13.3 ms, field of view, 160 × 160 mm, and matrix 208 × 166), sagittal proton density-weighted fat-suppression sequence (repetition time 2000 ms, echo time 15 ms, slice thickness 4.0 mm, interslice gap 0.4 mm, echo spacing 15 ms, field of view 160 × 160 mm, and matrix, 224 × 224), coronal water-select excited cartilage three-fluid sequence (repetition time 20 ms, echo time 7.7 ms, slice thickness 3 mm, interslice gap 1.5 mm, field of view 160 × 160 mm, and matrix 304 × 304), and axial T2-weighted fast-field echo sequence (repetition time 439.2 ms, echo time 13.8 ms, slice thickness 4 mm, interslice gap 0.4 mm, field of view 140 × 140 mm, and matrix 256 × 256).

The MRI scans were read by an experienced musculoskeletal radiologist (MDC) who was unaware of participants’ characteristics as well as clinical and radiographic data. Semi-quantitative assessments of the periarticular lesions were performed using the Whole-Organ MRI Score (WORMS) [9]. Baker’s cyst was scored from 0 to 3 (0 = none, 1 = small, 2 = medium, and 3 = large). Anserine and prepatellar bursitis, and tibiofibular cysts were scored as 0 or 1 (0 = none, 1 = present). A random subset of images (*n* =38) was re-read to determine intra-observer reproducibility (κ = 1.00 (1.00–1.00) for anserine bursitis, 0.64 (0.15–1.00) for prepatellar bursitis, 0.91 (0.72–1.00) for Baker’s cyst, and 1.00 (1.00–1.00) for tibiofibular cyst).

### Statistical analysis

We calculated the prevalence of periarticular lesions according to the presence of radiographic knee OA or knee pain. The prevalence of periarticular lesions in subjects with knee OA or knee pain was compared to those without using a chi-square test. All tests were two-tailed, and *P*-values < 0.05 were considered to indicate statistical significance. Analyses were performed using the SAS software (ver. 9.1; SAS Institute, Cary, NC, USA).

## Results

Among the 358 participants, 10 patients were excluded in the data analysis because only unilateral knee radiographs were taken due to a clerical error. Among 348 participants, the mean age was 72 years, and 50.6 % were female (Table 1). Radiographic OA and pain in the scanned knee were present in 34.5 % (14.5 % in males, 54.0 % in females) and 42.0 % (25.6 % in males, 58.0 % in females), respectively.

**Table 1:** Demographic and clinical characteristics of the subjects

	Subjects (*n* = 348)
Age, mean ± SD years	71.7 ± 5.8
Female	176 (50.6)
BMI, mean ± SD kg/m^2^	27.3 ± 52.4
Manual occupation	72 (20.7)
Radiographic knee OA	120 (34.5)
Knee pain in the last month	146 (42.0)

Values are number (percentage) of subjects, unless otherwise indicated

*SD* standard deviation, *BMI* body mass index, *OA* osteoarthritis

Periarticular lesions were present in 45.4 % of all participants (Table 2). The most prevalent lesion was a Baker’s cyst (27.9 %), followed by a tibiofibular cyst (9.5 %) and anserine bursitis (7.5 %). Periarticular lesions were present in 66.7 and 33.3 % of participants with and without knee OA, respectively. Anserine bursitis and tibiofibular cyst were significantly more common in the participants with knee OA than in those without it. Women had a higher prevalence of anserine bursitis (12.5 % in women, 1.7 % in men, *p* < 0.0001) and tibiofibular cysts than men (14.2 % in women, 4.1 % in men, *p* =0.0001) (data not shown).

**Table 2:** Prevalence of periarticular lesions according to the presence of radiographic knee OA

	OA (*n* = 120)	Non-OA (*n* = 228)	*P*-value
Baker’s cyst	39 (32.5)	58 (25.4)	0.163
Tibiofibular cyst	19 (15.8)	14 (6.1)	0.003
Anserine bursitis	21 (17.5)	5 (2.2)	<0.001
Prepatellar bursitis	2 (1.7)	0 (0.0)	0.118

Data presented are number of lesions (percentage)

*OA* osteoarthritis

Next, the prevalence of periarticular lesions according to the presence of knee pain was examined. (Table 3). Periarticular lesions were present in 60.1 and 33.5 % of participants with and without knee pain, respectively. Baker’s cyst and anserine bursitis were present in 34.6 and 14.4 % of participants with knee pain, respectively, which was significantly more prevalent compared to those without.

**Table 3:** Prevalence of periarticular lesions according to the presence of knee pain in the last month

	Knee pain (*n* = 146)	No knee pain (*n* = 202)	*P*-value
Baker’s cyst	53 (36.3)	44 (21.8)	0.003
Tibiofibular cyst	16 (11.0)	17 (8.4)	0.697
Anserine bursitis	21 (14.4)	5 (2.5)	<0.001
Prepatellar bursitis	2 (1.4)	0 (0.0)	0.244

Data presented are number of lesions (percentage)

*OA* osteoarthritis

## Discussion

Periarticular lesions were common, affecting 45 % of these community-based older adults. The most prevalent lesion was Baker’s cyst (27.9 %), followed by tibiofibular cyst and anserine bursitis. Periarticular lesions were more prevalent in subjects with OA than in those without OA. Anserine bursitis and tibiofibular cyst were more common in subjects with knee OA, while Baker’s cyst and anserine bursitis were more common in subjects with knee pain.

Benign cystic lesions are frequently seen on MRI of the knee. Baker’s cysts, the most frequently encountered, is not a true cyst, but a fluid collection in the semi-membranosus-medial gastrocnemius bursa. MRI studies have shown that the prevalence of Baker’s cysts is as high as 40 % [4, 10, 11]. There is conflicting data regarding the association of Baker’s cysts with radiographic knee OA. Although a higher prevalence of Baker’s cyst was reported in those with than without knee OA [10, 12], others reported no difference in its prevalence, consistent with our result [13]. In people with frequent chronic pain, no trend was observed between its presence and increasing radiographic knee OA severity [11].

Tibiofibular cysts, which are caused by an increase in intra-articular pressure due to active synovitis or joint injury, cause an outpouching of the tibiofibular joint capsule, herniating to form the synovial cyst [11]. Although we found that tibiofibular cysts were more common in subjects with knee OA, previous results did not reveal significant association between tibiofibular cysts and knee OA after adjustment of confounders [11, 14].

Chronic anserine bursitis, a common lesion in elderly patients who have OA or rheumatoid arthritis [15], was reported not to be significantly associated with knee OA in two recent case–control studies [14, 16]. On the other hand, our study showed higher prevalence of anserine bursitis in knee OA subjects, and the discrepancy may be due to differences in the populations studied or the detection limits. Also, because only a small number of lesions were available for analysis, further longitudinal studies with a larger sample size will be helpful in this regard.

In this study, women had a higher prevalence of anserine bursitis and tibiofibular cysts than men. These results may be in part related to a higher prevalence of knee OA among women and other multifactorial factors.

Because cartilage is avascular and aneural, the pain in OA is not likely to result from cartilage damage *per se*, and may arise from structures outside the joint cavity [2, 17, 18]. We found that Baker’s cyst and anserine bursitis were more prevalent in subjects with knee pain. In previous studies, Baker’s cyst was also associated with weight-bearing pain and painful flare in OA patients [5, 19]. However, other reports have shown no relationship between Baker’s cyst, anserine bursitis, and pain in people with OA [10, 14, 20]. The presence of Baker’s cyst in knees with osteoarthritic pain has been shown to correlate with synovial inflammation [12]. Inflammation from various local and systemic processes, such as overuse, trauma, infection, hemorrhage, internal joint derangement, and inflammatory arthropathy, leads to thickening of the synovial lining and fluid accumulation within the bursae around the knee joint [15]. Acute anserine bursitis results frequently from overuse injury or trauma, especially in runners, causing medial knee pain, whereas chronic anserine bursitis occurs in patients with degenerative joint disease or rheumatoid arthritis [15, 16, 21]. Knee pain arising from it may be determined by multiple sources, including not only structural damage, pain processing mechanism, and gender, but also activities of daily living provoking the pain, which may depend on cultural differences among the populations studied.

This study has several limitations. First, only anterio-posterior knee radiographs were obtained, preventing the evaluation of patellofemoral OA. Second, because we did not have surgical or pathological confirmation, some of the lesions, such as bursitis, may have been a simple collection of fluid without inflammation. Third, periarticular lesions other than the ones included were found infrequently, so that we could not subject them to statistical analyses. Finally, although we examined cartilage morphology including signal change and thickness, we did not measure cartilage volume in our study, which could be a limitation in the data analysis.

## Conclusions

This study is the first reported population-based research using MRI to investigate the relationships between knee pain and periarticular lesions in Asian subjects. Because baker’s cyst and anserine bursitis were significantly more common in subjects with knee pain, specific treatment of this lesion in addition to systemic analgesic treatment might further improve pain in knee OA patients.

### Availability of data and materials

Data will not be shared because of the policy of the Hallym Aging Study principal investigator (DH Kim).

## Abbreviations

BMI: body mass index
K-L: Kellgren-Lawrence
MRI: magnetic resonance imaging
OA: osteoarthritis
WOMAC: Western Ontario and McMaster Universities
WORMS: Whole-Organ MRI Score
